# Case Report: Ischemic brain infarction and cognitive dysfunction syndrome in an aged dog

**DOI:** 10.3389/fvets.2025.1563798

**Published:** 2025-03-19

**Authors:** Min-Hee Kang, Woo-Phil Jeong, Chan-Sik Nam, Jun-Won Yoon, Dong-Min Choi, Gwang-Seob Lee, Yeon-Jin Kim, Tae-Jung Dan, Hee-Myung Park

**Affiliations:** ^1^Department of Bio-Animal Health, Jangan University, Gyeonggi-do, Republic of Korea; ^2^Gangnam Annie Animal Hospital, Gyeonggi-do, Republic of Korea; ^3^Department of Veterinary Internal Medicine, College of Veterinary Medicine, Konkuk University, Seoul, Republic of Korea

**Keywords:** dog, geriatric, cognitive dysfunction syndrome, brain infarction, cerebrovascular pathology

## Abstract

This case report describes a rare occurrence of canine cognitive dysfunction syndrome (CDS) accompanied by ischemic brain infarction, providing insights into the relationship between neurodegeneration and cerebrovascular pathology in aged dogs. A 19-year-old neutered male miniature poodle exhibited progressive behavioral changes over three years, including nocturnal restlessness, inappropriate urination, and aimless wandering. Neurological examination revealed mild disorientation, decreased proprioception, and weakened postural reactions in the hind limbs, with a cognitive dysfunction rating (CDDR) score of 64 indicating severe cognitive impairment. Magnetic resonance imaging (MRI) revealed hallmark indicators of brain atrophy, such as widened cerebral sulci and ventricular enlargement, along with multifocal ischemic lesions in the right parietal and occipital area. Histopathological findings confirmed widespread neurodegeneration, including severe vacuolation and neuronal necrosis in the precentralis interna and anterior subcallosal regions. Congo Red-positive staining identified amyloid-like deposits in cerebral vessels, and Lewy bodies in the brainstem suggested concurrent vascular and amyloid pathology. This case provides evidence of a potential connection between cerebrovascular pathology and CDS, indicating that ischemic and hemorrhagic lesions may aggravate neurodegeneration and contribute to cognitive and neurological deficits. The coexistence of brain infarction and amyloid deposits in this dog resembles pathological processes observed in human conditions such as Alzheimer’s disease and vascular dementia, highlighting the multifactorial nature of CDS. Advanced neuroimaging and histopathological analysis were critical in diagnosing and understanding this complex interaction. Further research is needed to clarify the mechanisms linking neurodegeneration and cerebrovascular disease in aging dogs.

## Introduction

Canine cognitive dysfunction syndrome (CDS) is a neurodegenerative disorder observed in aging dogs, often compared to Alzheimer’s disease (AD) in humans due to similar clinical signs and neuropathological features (1, 2). CDS primarily affects dogs older than eight years, leading to progressive cognitive decline, behavioral abnormalities, and memory impairment, closely resembling human dementia (3). These similarities have positioned CDS as a valuable translational model for studying neurodegenerative diseases, contributing to a better understanding of disease mechanisms across species (2, 4). However, CDS remains underdiagnosed, resulting in many untreated cases and significantly affecting the quality of life for both affected dogs and their caregivers.

Despite these similarities, significant pathological distinctions exist. While both CDS and AD involve neuronal degeneration and vascular changes, hallmark features of AD, such as neurofibrillary tangles and Hirano bodies, are absent in dogs with CDS (1, 2, 5, 6). Nevertheless, studies indicate that tau hyperphosphorylation and amyloid-*β* accumulation in the aging canine brain may contribute to cognitive impairment, reinforcing the need for further research into shared and distinct pathological mechanisms (6, 7).

Cerebrovascular pathology, including brain infarction, is uncommon in dogs and has rarely been documented alongside CDS (8). However, recent studies suggest that neuroinflammation, oxidative stress, and vascular dysfunction may contribute to both neurodegenerative and cerebrovascular diseases, underscoring the importance of evaluating their potential interplay (7, 9, 10). Investigating these connections may improve our understanding of their impact on aging animals and guide clinical management.

This case report presents a rare occurrence of CDS accompanied by brain infarction in a geriatric dog. Imaging and histopathological analyses were conducted to explore the potential relationship between cognitive dysfunction and cerebrovascular pathology, offering insights into similar conditions in aging animals.

## Case description

### Case presentation and diagnostic investigations

A 19-year-old neutered male miniature poodle, weighing 5.2 kg, was presented with progressive behavioral changes, including nocturnal restlessness, excessive barking, aimless wandering at night, and compulsive pacing. Additional clinical signs included inappropriate urination and intermittent oral bleeding. The owner reported a gradual progression of these clinical signs over three years, leading to concerns about the dog’s overall health and the possibility of CDS.

On physical examination, the dog appeared lethargic and less responsive to environmental stimuli. Pale mucous membranes were noted, and a firm mass was palpable in the right maxillary region. Mild respiratory distress was observed, with a respiratory rate of 40 breaths per minutes. Additionally, a suspected mammary gland tumor was identified in the left fourth mammary gland. Other physical examination findings were unremarkable. The neurological status of the dog was evaluated. The dog showed mild disorientation and slightly decreased proprioception in all limbs. The cranial nerve examination was unremarkable, and postural reactions were mildly weakened in the hind limbs. The spinal reflexes were within normal limits.

An assessment for CDS was conducted based on the dog’s medical history, clinical signs, physical examination and neurological findings. The dog scored 64 points on the canine cognitive dysfunction rating scale (CCDR) (11), indicating severe cognitive impairment.

Hematological evaluation revealed severe anemia [hematocrit (HCT) 13.5%, reference interval (RI) 37.3–61.7%; red blood cell count (RBC) 1.79 × 10^12/L, RI 5.65–8.87 × 10^12/L; hemoglobin (HGB) 4.1 g/dL, RI 13.1–20.5 g/dL] and marked leukocytosis [white blood cell count (WBC) 30.75 × 10^9/L; RI 5.05–16.76 × 10^9/L]. Reticulocytosis was significant (333.6 × 10^3/μL; RI 10–110 × 10^3/μL). Biochemical analyses showed mild elevations in symmetric dimethylarginine (SDMA) levels (15 μg/dL; RI ≤ 14 μg/dL), alanine aminotransferase (57 U/L; RI 0–50 U/L) and alkaline phosphatase (1,036 U/L; RI 23–212 U/L), alongside markedly elevated C-reactive protein (50.8 mg/dL; RI 0–10 mg/dL). Electrolyte levels were within normal limits.

Diagnostic imaging was performed to assess the maxillary and mammary masses and evaluate systemic involvement. Computed tomography (CT) of the head revealed a 2.4 × 0.8 × 4.5 cm mass in the right maxilla, with extensive alveolar bone resorption affecting the maxillary premolars and molars, while the medial cortical bone remained intact. CT of the left mammary region identified a calcified mass (1.9 × 2.9 × 3.0 cm) suggestive of a subcutaneous mammary gland tumor. No evidence of metastasis was observed.

**Figure 1:**
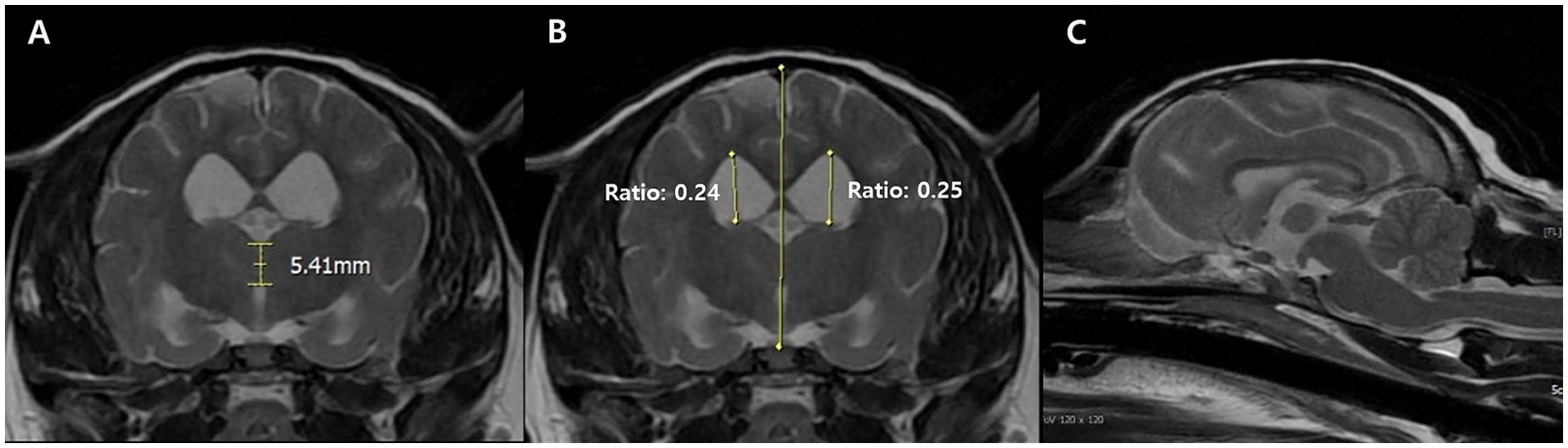
Transverse T2-weighted magnetic resonance images of CDS dog at the level of the interthalamic adhesion. The measurement of the interthalamic adhesion was 5.41 mm and heights of the right and left ventricles (ventricle to brain height ratio) is measured at 24–25% in the transverse view **(A,B)**. Generalized broadened and deeply observed cerebral sulci, along with ventricular enlargement was also prominent at the sagittal view **(C)**.

**Figure 2:**
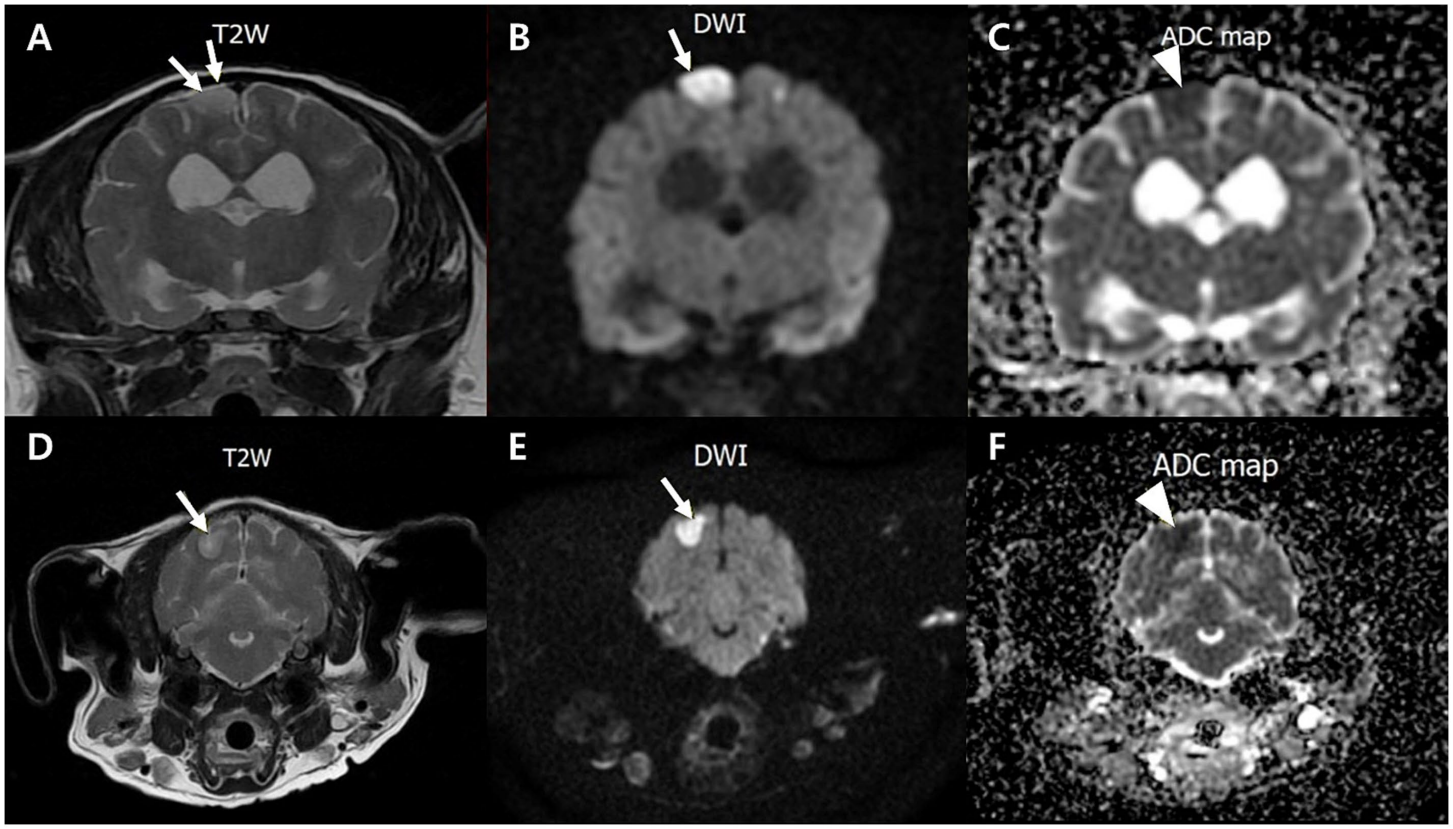
Transverse T2-weighted, diffusion-weighted imaging (DWI) and an apparent diffusion coefficient (ADC) magnetic resonance images of a dog with multifocal brain infarcts. Lesion locates in the right parietal **(A–C)** and occipital area **(D–F)** appears hyperintense with peripheral hypointensity (arrows) on T2 **(A,D)** and DWI **(B,E)**, and hypointensity (arrowhead) on an ADC map **(C,F)**.

Neurological signs prompted magnetic resonance imaging (MRI), which revealed widened cerebral sulci and ventricular enlargement, consistent with brain atrophy. The ventricle-to-brain height ratio (VBHR) was 24–25%, exceeding the normal threshold (< 15%), indicating ventricular dilation. The interthalamic adhesion (ITA) measured 5.41 mm, which is lower than the normal range (6.09–7.49 mm) but thicker than the range typically observed in dementia (3.03–4.61 mm) (25). (Figure 1). T2-weighted and diffusion-weighted imaging (DWI) demonstrated multifocal hyperintensities in the right parietal and occipital area, located in the dorsolateral regions of the cerebrum, consistent with ischemic lesions. Apparent diffusion coefficient (ADC) mapping confirmed restricted diffusion, indicative of brain infarction (Figure 2). These findings were suggestive of cerebrovascular abnormalities contributing to cognitive dysfunction and neurological deficits.

Given the severity of the clinical conditions, including advanced CDS and cerebrovascular disease, humane euthanasia was elected. A limited post-mortem examination focusing on the central nervous system was performed to confirm the imaging findings and obtain a comprehensive understanding of the underlying pathology.

**Figure 3:**
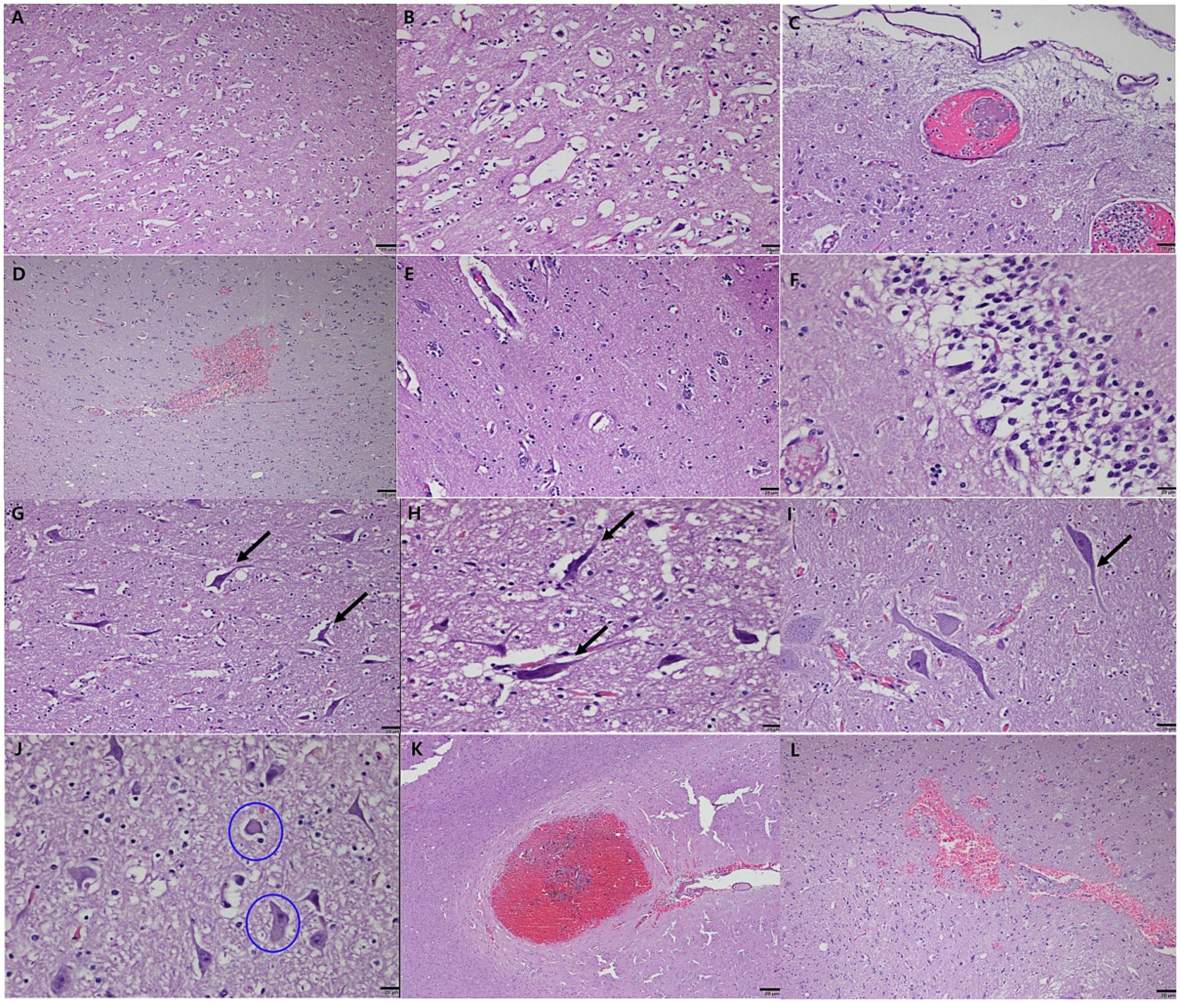
Histopathological features of neurodegeneration and cerebrovascular pathology in a geriatric dog with CDS. Severe vacuolation and neuronal necrosis in the precentralis interna and anterior subcallosal region of the cerebral cortex **(A,B)**. Low magnification view showing extensive vacuolation **(A)**. High magnification view highlighting pronounced neuronal necrosis **(B)**. Intravascular microthrombi distributed in the perivascular spaces **(C)** and microhemorrhagic infarcts **(D)** in the right parietal lobe, consistent with ischemic brain injury. Reactive gliosis with numerous microglia and astrocytes closely associated with neurons, observed in the surrounding cortical areas **(E)**. Pyknotic neurons and neurofibrillary tangle-like structures in the CA (Cornu Ammonis) 1 and CA2 regions of the hippocampus, resembling features of human neurodegenerative diseases **(F)**. Axonal degeneration and neurofibrillary tangle-like structures in the brainstem **(G)** and posterior cerebellum **(H,I)**. Lewy bodies (circled) identified within neuronal cells in the brainstem, indicative of abnormal protein aggregation **(J)**. Hemorrhagic infarcts in the inferior frontal gyrus **(K)** and subcallosal region of the anterior cerebral hemisphere **(L)**. Note: Scale bar = 20 μm for all panels.

**Figure 4:**
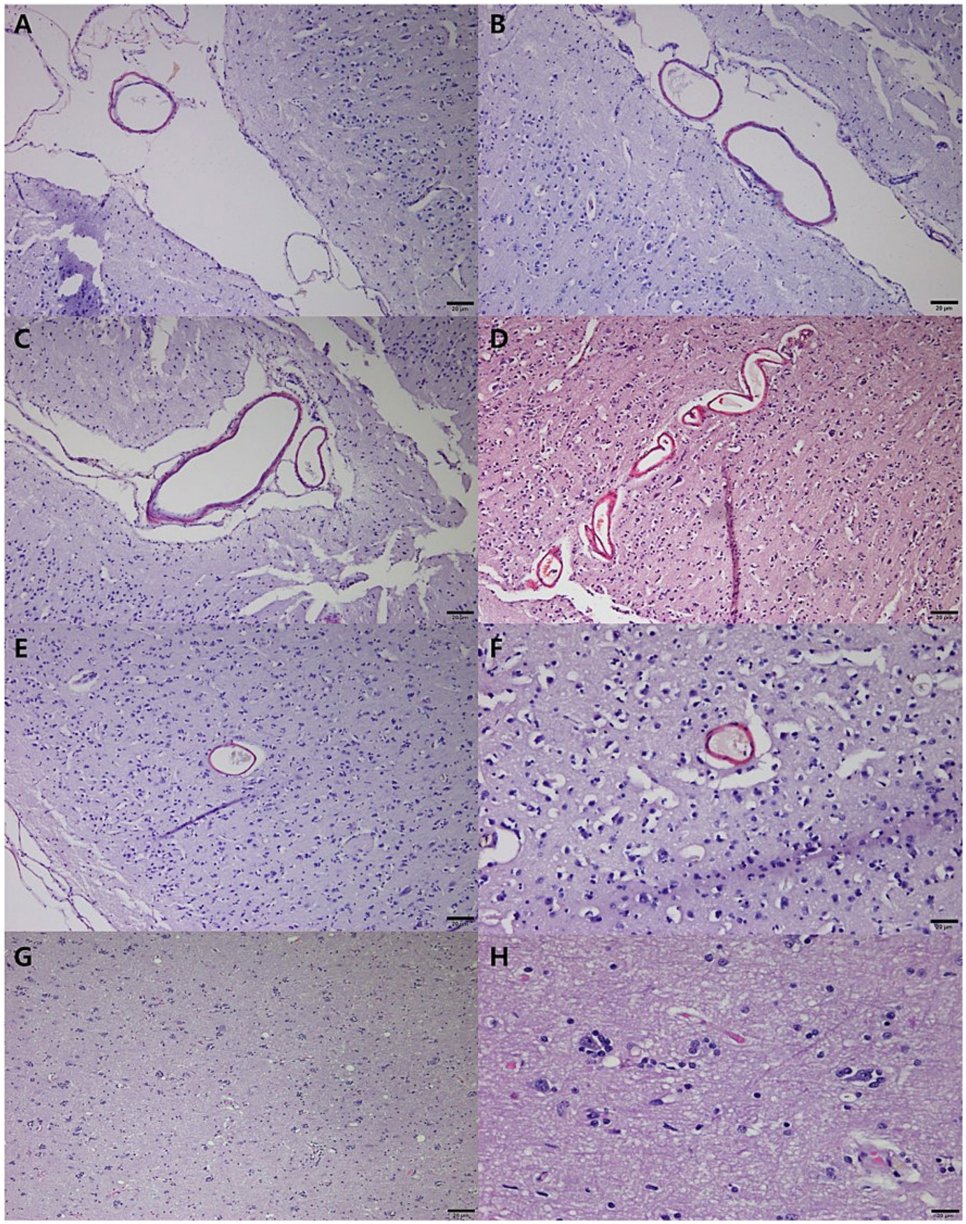
Congo red staining of neurodegeneration and cerebrovascular pathology in a geriatric dog with CDS. Congo Red-positive vascular deposits in veins within less folded sulci on the cortical surface of the frontal area **(A,B)**. Amyloid-like deposits in vessels of the prefrontal cortex, particularly in sulci on the cortical surface **(C,D)**. Congo Red positivity in vessels of the marginal gyrus in the anterior **(E)** and posterior **(F)** cerebral cortices, with thickened vessels surrounding neurons. Reactive gliosis in the frontal gyrus near the caudate nucleus, characterized by increased astrocytes and microglia **(G,H)**. Scale bar = 20 μm for all panels.

Histopathological analysis revealed extensive neurodegeneration, with widespread vacuolation and neuronal necrosis prominently affecting the precentralis interna and anterior subcallosal regions (Figures 3A,B). In addition, the right parietal regions, corresponding to the hyperintensity areas observed on MRI, revealed intravascular microthrombi distributed in the perivascular spaces and microhemorrhagic infarcts in the surrounding cortical areas, consistent with ischemic brain injury (Figures 3C,D). Numerous microglia and astrocytes were closely associated with neurons, indicating reactive gliosis (Figure 3E). Pyknotic neurons and structures resembling neurofibrillary tangles were observed in the hippocampus (Figure 3F) and brainstem (Figures 3G–I). Lewy body-like structures were identified in the brainstem (Figure 3J), suggesting the possibility of abnormal protein aggregation. Additionally, vascular abnormalities included hemorrhagic infarcts in the inferior frontal gyrus (Figure 3K) and subcallosal regions (Figure 3L). Congo Red-positive staining confirmed amyloid-like vascular deposits in the frontal (Figures 4A,B), prefrontal (Figures 4C,D), and cerebellar cortices (Figures 4E,F). Reactive gliosis, with increased astrocytes and microglia, was observed in the frontal gyrus near the caudate nucleus (Figures 4G,H). These findings supported a diagnosis of advanced neurodegenerative disease with concurrent cerebrovascular pathology.

## Discussion

This case report describes a rare presentation of CDS accompanied by ischemic brain infarction, providing insights into the relationship between neurodegeneration and cerebrovascular pathology in aged dog. While neurodegenerative diseases such as CDS and AD share several clinical and pathological features, this report emphasizes the distinctive aspects of CDS and its association with brain infarcts.

Over three years, the dog exhibited hallmark signs of cognitive dysfunction, including nocturnal restlessness, inappropriate urination, and behavioral changes. These clinical features aligned with a severe CDS diagnosis, confirmed by a CDDR score of 64. Canine CDS and human AD share common features, such as cognitive decline and behavioral changes (12, 13). However, significant pathological differences exist between the two. Prominent neurofibrillary tangles, a hallmark of AD, are notably less prominent in CDS, suggesting differences in the underlying mechanisms (12, 14–16).

The findings of this case provide evidence for the role of cerebrovascular pathology in the progression of CDS. MRI findings demonstrated hallmark indicators of brain atrophy, including widened cerebral sulci and ventricular enlargement, consistent with neural tissue loss due to aging and chronic disease (14, 17–19). In addition, diffusion-weighted imaging identified multifocal ischemic lesions in the right parietal and occipital areas, emphasizing the role of cerebrovascular events in the disease progression (8, 20, 21). These ischemic changes, combined with hemorrhagic infarcts, created a complex pathology exacerbating the dog’s cognitive and neurological deficits (6). Similar observations have been reported in dogs with brain infarcts, indicating significance of vascular alterations in neurodegenerative conditions (4, 21). In particular, the presence of ischemic and hemorrhagic lesions has been shown to exacerbate neurodegenerative processes, further impairing cognitive function in both dogs and humans (10, 17). This case emphasizes the role of cerebrovascular pathology in the progression of CDS and reflects similarities with pathological processes seen in human conditions, such as vascular dementia and AD.

Histopathological findings confirmed widespread neurodegeneration, including severe vacuolation and neuronal necrosis in the precentralis interna and anterior subcallosal regions. Pyknotic neurons and structures resembling neurofibrillary tangle-like inclusions were identified in the hippocampus and brainstem. Although these inclusions were morphologically consistent with neurofibrillary tangles, definitive confirmation was not possible due to the absence of silver impregnation or immunohistochemical (IHC) staining for phosphorylated Tau. Additionally, eosinophilic cytoplasmic inclusions resembling Lewy bodies were observed, suggesting the possibility of abnormal protein aggregation. However, the possibility of lipofuscin accumulation could not be excluded, highlighting the need for confirmatory IHC staining, such as ubiquitin or alpha-synuclein, in future studies (9, 14). Amyloid-like deposits in the vessels of the frontal, prefrontal, and cerebellar cortices, verified through Congo Red-positive staining, suggest a role for amyloid angiopathy in the development of CDS (1, 4). The identification of amyloid deposits and reactive gliosis supports the hypothesis that vascular amyloid pathology plays a contributory role in cognitive dysfunction (10, 22). Such findings are consistent with studies demonstrating that dogs with CDS frequently exhibit amyloid deposition in the brain parenchyma and vasculature, suggesting shared mechanisms with AD (1, 4, 22).

The vascular abnormalities observed in this case are particularly significant and provide valuable insights into the underlying mechanisms contributing to the dog’s clinical deterioration. Hemorrhagic infarcts were identified in the inferior frontal gyrus and subcallosal regions in this dog, strongly indicating a substantial cerebrovascular component to the observed neurological and cognitive decline. These vascular lesions likely disrupted critical neural networks responsible for cognition and behavior by reducing regional cerebral perfusion and contributing to microvascular pathology, such as blood–brain barrier dysfunction and neuronal damage (9, 22). Neuropathological evidence suggests that cerebrovascular alterations, including silent brain infarcts, exacerbate pre-existing neurodegenerative processes, thereby accelerating cognitive and behavioral deterioration (9, 23). Silent brain infarcts, which are linked to increased dementia risk and accelerated cognitive decline in humans, may exert similar effects in dogs, underscoring the importance of vascular factors in CDS progression (23).

This case is distinguished by its integration of imaging and histopathological findings to demonstrate the relationship between cerebrovascular disease and neurodegeneration. While prior studies have documented microhemorrhages and brain atrophy in aging dogs (15, 18), few have explored the association between ischemic infarcts and CDS. In human dementia research, the link between silent brain infarction and AD is well established, but similar studies in dogs are rare (9, 10, 23, 24). This report contributes to the field by proposing a potential connection between brain infarction and CDS, using advanced diagnostic methods to support the findings.

However, several limitations should be noted. The absence of confirmatory IHC staining limited the definitive characterization of key neuropathological findings. Structures resembling neurofibrillary tangles were identified in the hippocampus and brainstem but could not be conclusively distinguished without silver impregnation or IHC for phosphorylated Tau. Similarly, eosinophilic cytoplasmic inclusions resembling Lewy bodies were observed in the brainstem, suggesting abnormal protein aggregation. However, the possibility of lipofuscin accumulation could not be excluded. Reactive gliosis was identified based on astrocytic and microglial proliferation observed on H&E staining. However, the absence of IHC markers such as GFAP for astrocytes and Olig2 for oligodendroglial lineage cells limited the precise characterization of gliosis and neuroinflammation. Additionally, the absence of serial imaging and longitudinal clinical monitoring limits the ability to establish a definitive temporal relationship between the vascular events and the progression of cognitive dysfunction.

These limitations emphasize the need for advanced diagnostic techniques, including IHC and special staining, to improve diagnostic accuracy and provide a more comprehensive understanding of neurodegenerative and cerebrovascular changes in CDS. Future studies employing these advanced methods are essential to clarify the relationship between neurodegenerative and vascular changes and to develop more effective diagnostic and therapeutic strategies for aging dogs with CDS.

In conclusion, this case illustrates the multifactorial nature of CDS, with both neurodegenerative and vascular factors contributing to the dog’s clinical presentation. The coexistence of cognitive decline, ischemic infarcts, and amyloid deposits suggests that CDS may represent a complex interaction between neurodegeneration and cerebrovascular pathology.

## Data Availability

The original contributions presented in the study are included in the article/supplementary material, further inquiries can be directed to the corresponding author/s.
